# Trans-epithelial migration is essential for neutrophil activation during RSV infection

**DOI:** 10.1093/jleuko/qiad011

**Published:** 2023-02-02

**Authors:** Elisabeth Robinson, Jenny Amanda Herbert, Machaela Palor, Luo Ren, Isobel Larken, Alisha Patel, Dale Moulding, Mario Cortina-Borja, Rosalind Louise Smyth, Claire Mary Smith

**Affiliations:** Infection, Immunity and Inflammation Department, UCL Great Ormond Street Institute of Child Health, 30 Guilford Street, London WC1N1EH, United Kingdom; Infection, Immunity and Inflammation Department, UCL Great Ormond Street Institute of Child Health, 30 Guilford Street, London WC1N1EH, United Kingdom; School of Medical Sciences, Faculty of Biology, Medicine, and Health, University of Manchester, Oxford Rd, Manchester M13 9PL, United Kingdom; Infection, Immunity and Inflammation Department, UCL Great Ormond Street Institute of Child Health, 30 Guilford Street, London WC1N1EH, United Kingdom; Infection, Immunity and Inflammation Department, UCL Great Ormond Street Institute of Child Health, 30 Guilford Street, London WC1N1EH, United Kingdom; Department of Respiratory Medicine, Children's Hospital of Chongqing Medical University, Chongqing 400014, China; Infection, Immunity and Inflammation Department, UCL Great Ormond Street Institute of Child Health, 30 Guilford Street, London WC1N1EH, United Kingdom; Infection, Immunity and Inflammation Department, UCL Great Ormond Street Institute of Child Health, 30 Guilford Street, London WC1N1EH, United Kingdom; Infection, Immunity and Inflammation Department, UCL Great Ormond Street Institute of Child Health, 30 Guilford Street, London WC1N1EH, United Kingdom; Infection, Immunity and Inflammation Department, UCL Great Ormond Street Institute of Child Health, 30 Guilford Street, London WC1N1EH, United Kingdom; Infection, Immunity and Inflammation Department, UCL Great Ormond Street Institute of Child Health, 30 Guilford Street, London WC1N1EH, United Kingdom; Infection, Immunity and Inflammation Department, UCL Great Ormond Street Institute of Child Health, 30 Guilford Street, London WC1N1EH, United Kingdom

**Keywords:** virus infection, bronchiolitis, airway epithelial cells, migration, movement, neutrophils

## Abstract

The recruitment of neutrophils to the infected airway occurs early following respiratory syncytial virus (RSV) infection, and high numbers of activated neutrophils in the airway and blood are associated with the development of severe disease. The aim of this study was to investigate whether trans-epithelial migration is sufficient and necessary for neutrophil activation during RSV infection.

Here, we used flow cytometry and novel live-cell fluorescent microscopy to track neutrophil movement during trans-epithelial migration and measure the expression of key activation markers in a human model of RSV infection. We found that when migration occurred, neutrophil expression of CD11b, CD62L, CD64, NE, and MPO increased. However, the same increase did not occur on basolateral neutrophils when neutrophils were prevented from migrating, suggesting that activated neutrophils reverse migrate from the airway to the bloodstream side, as has been suggested by clinical observations. We then combined our findings with the temporal and spatial profiling and suggest 3 initial phases of neutrophil recruitment and behavior in the airways during RSV infection; (1) initial chemotaxis; (2) neutrophil activation and reverse migration; and (3) amplified chemotaxis and clustering, all of which occur within 20 min. This work and the novel outputs could be used to develop therapeutics and provide new insight into how neutrophil activation and a dysregulated neutrophil response to RSV mediates disease severity.

## Teaser

Building on previous work of neutrophil function, we propose 3 main phases of early neutrophil recruitment and behavior in the airways during RSV infection. Phase 1: Initial chemotaxis and adherence: here unstimulated circulating neutrophils expressing baseline levels of CD11b migrate across infected AECs in response to chemotactic signals in the apical supernatant. Some neutrophils remain adherent to the infected AECs. Phase 2: Activation and reverse migration: once on the apical side of the epithelium, neutrophils increase expression of CD11b and other activation-associated markers, and some “activated” neutrophils undergo reverse migration. Neutrophils with greater expression of CD11b are detected on the basolateral side. Phase 3: Amplified chemotaxis and clustering: after 20 min, adherent neutrophils begin to rapidly cluster on RSV-infected primary airway epithelial cells cultures, mediated by signaling from a dying neutrophil. Drawing created using BioRender.com.

## 1 Introduction

Respiratory syncytial virus (RSV) is a seasonal respiratory virus, reported to infect almost all children before the age of 2.^1^ Following infection, most children develop an illness that is confined to upper respiratory tract symptoms; however, 1% to 3% of infected infants will develop a severe illness requiring hospitalization.^2^ There is no licensed vaccine for RSV, and treatment is currently limited to supportive care. In resource-limited countries, RSV is a major cause of infant mortality.^3–5^

Although risk factors have been identified, it is still not clear why RSV-infected infants, with no apparent risk factors, require hospitalization and respiratory support.^6^ One suggestion is that neutrophils, which form around 80% of all cells recovered from the airways of infants with severe RSV bronchiolitis by bronchoalveolar lavage,^7^ and their associated cytokines contribute to disease severity.^8–10^ Neutrophil-mediated factors such as CXCL8 (IL-8), CXCL10 (IP-10), and neutrophil elastase are also present in substantial amounts in the airway secretions of children with severe RSV bronchiolitis.^11–13^

Neutrophils are the predominant immune cell type in the systemic circulation and are the first cell recruited from the bloodstream through the airway epithelium during infection.^14^ Here they are thought to employ largely nonspecific mechanisms of pathogen destruction, including phagocytosis, neutrophil extracellular trap formation, and release of toxic granule products such as neutrophil elastase and myeloperoxidase.^15,16^ Neutrophil trans-epithelial migration is facilitated by neutrophil receptors including integrins (i.e. CD11b) and selectins (such as CD62L) that bind to molecules on airway epithelial cells such as ICAM-1.^17–19^ Neutrophils also interact with other immune complexes using Fc receptors such as CD64; upregulation of CD64 has been evaluated as a biomarker for neonatal sepsis in infants.^20^

Several in vitro and in vivo systems have been developed to study neutrophil migration, including airway and intestinal epithelial models.^21,22^ We have developed an RSV infection model using primary differentiated airway epithelial cells and found that neutrophil migration resulted in increased epithelial damage and a reduced viral load.^23,24^ We observed that neutrophils adhere to RSV-infected AECs in a clustering pattern, which was not seen in uninfected AECs. This pattern of neutrophil chemotaxis and migration has been reported using in vivo models, referred to as “neutrophil swarming” due to a resemblance with the swarming behavior of insects.^25,26^ We also showed that migrated neutrophils have greater cell surface expression of CD11b and MPO compared to neutrophils that had not migrated.^27^ What remains unclear is whether migrated neutrophils with higher CD11b, for example, are selected for trans-epithelial migration or whether they become activated due to migration per se. Although RSV is not known to establish infection outside the respiratory tract or cause a viraemia, clinical studies have shown that a high proportion of neutrophils from the systemic circulation of infants with RSV bronchiolitis contain RSV mRNA.^11^ This raises the possibility that activated neutrophils, recruited to the airway during RSV infection, may be able to migrate in the reverse direction, back to the systemic circulation.

**Fig. 1.**
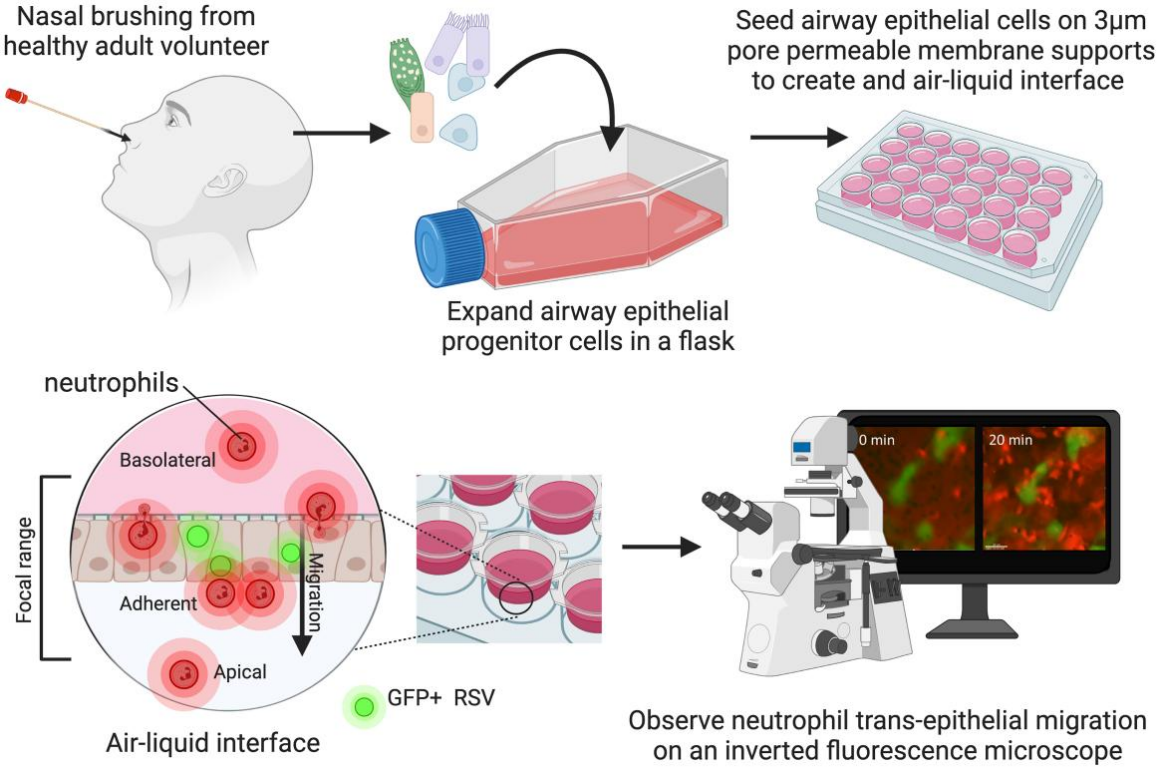
Schematic of method and model used to study neutrophil trans-epithelial migration in response to RSV infection. Human primary airway epithelial cells (AECs) were cultured on the underside of a 3 μm pore size, transparent PET culture membrane insert. AECs were matured at an air–liquid interface (ALI) for 7 d before infection with GFP tagged RSV. At this time, neutrophils, stained with a viability stain (calcein-red orange), were added to the basolateral side of AEC cultures, and a 50 μm Z-stack image of the focal area indicated was captured for up to 1 h. Drawing created using Biorender.com.

This study investigates the behavior and function of neutrophils as they move across the airway epithelial cell layer during RSV infection. We studied this migration using a human model of primary airway epithelial cells grown at the air–liquid interface for 7 d. This model facilitated the higher-resolution z-stack microscopy needed for 4D (XYZT) tracking of neutrophil migration for the first time. (Figure 1). We used this model to evaluate the kinetics of neutrophil trans-epithelial migration, including “cluster formation,” and the temporal and spatial association of neutrophil activation during RSV infection.

## 2 Results

### 2.1 Neutrophil trans-epithelial migration increases damage to airway epithelial cells associated with a release of neutrophil proteases

First, as we have done before in differentiated cultures,^24^ we characterized whether neutrophil migration increases epithelial damage during RSV infection in our novel AEC model. As controls, we compared epithelial damage and neutrophil protease release across either (1) mock-infected AECs, (2) mock-infected AECs exposed to potent neutrophil chemoattractant (fMLP), or (3) mock-infected AECs exposed to RSV infected AEC supernatant (referred to as RSV Sup). These controls allowed us to differentiate whether our observations were due to the process of migration per se or to inflammatory mediators present in the RSV-infected airway supernatant.

**Fig. 2.**
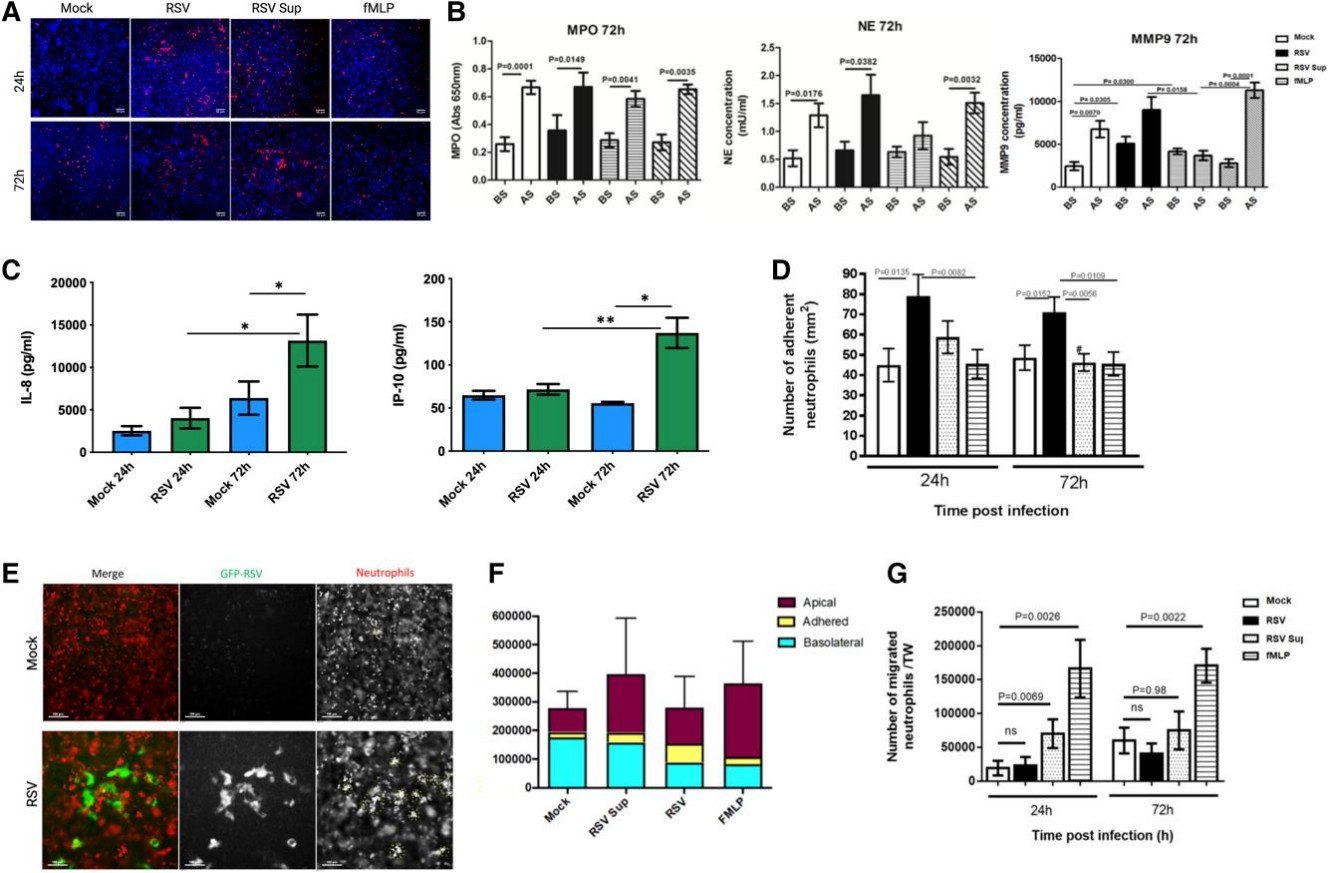
RSV increases the numbers of neutrophils that adhere to RSV-infected epithelial cultures, forming clusters on the AEC surface. (A) Representative image showing neutrophil (red cells) adherence to epithelial cells (DAPI staining—blue) following migration for 1 h at 24 h or 72 h after RSV or mock infection. fMLP 1 ng/ml placed apical to uninfected AECs was used a positive control for neutrophil chemotaxis. Scale bar indicates 50 µm (B) Soluble granular factors myeloperoxidase (MPO), neutrophil elastase (NE), and matrix-metalloproteinase-9 (MMP-9) in the apical supernatant (designated AS in figure) and basolateral supernatant (BS) were quantified using ELISA. fMLP and mock-infected AECs with supernatant collected from RSV-infected AECs placed apically (RSV Sup). (C) Concentration of IL-8 and IP-10 in the apical supernatants from RSV-infected AECs (green) or mock-infected AECs (blue) infected for 24 or 72 h. Groups were compared with 1-way ANOVA with Tukey's post hoc adjustment for multiple testing. * *P* < 0.05. ** *P* < 0.002. (D) Graph showing representative counts of neutrophils isolated either basolateral to, apical to, or adherent to AECs. Absolute counts performed using a flow cytometer selecting for neutrophils as positive for CD11b-APC antibody staining. (E) Representative 2-channel maximum intensity projection of a Z-stack image (50 µm range) of 72 h mock (top) and RSV (bottom) showing neutrophil adherence in clusters to mock- and RSV-infected AECs. Green = RSV-infected AECs, red = neutrophils. Scale bar indicates 100 µm. (F) Numbers of neutrophils adherent to AECs after 1 h transepithelial migration across 24 or 72 h RSV-infected AECs. Adherent neutrophils and epithelial cells were counted using ImageJ counting tool; the average number of neutrophils from all images is shown. (G) Numbers of neutrophils migrating and dissociating apically from AECs after 1 h transepithelial migration across 24 or 72 h RSV-infected AECs. Neutrophil concentrations were quantified in the apical surface media using a plate reader and read against a standard curve.

We found that 1 h after neutrophil trans-epithelial migration across epithelium infected with RSV for 24 h, we detected larger gaps with mean ± standard error of the mean (SEM) of 70.8 ± 4.6% area in the RSV-infected epithelial layer compared to the mock-infected (61.6 ± 6.0% area) (*P* < 0.001) (representative images shown in Fig. 2A). We also detected a loss of RSV-infected cells, as is observed in Supplementary Video 1. This video shows that as neutrophil transepithelial migration progresses, a GFP-expressing AEC (i.e. positive for replicating RSV) disappears from the image, indicating loss of infected cells following neutrophil migration. A comparative video of neutrophils migrating across mock-infected cells is shown in Supplementary Video 2.

Neutrophils release several toxic products, including myeloperoxidase (MPO) and neutrophil elastase (NE). We measured the concentration of these products in the compartments of our model after neutrophil migration. We found that the concentration of NE, in airway surface media of AEC cultures, was >3-fold greater than the basolateral media following neutrophil migration across RSV-infected epithelium for 4 h at 72 h postinfection (Fig. 2B) with a mean ± SEM of 2.0 ± 0.6 mU/ml, compared to 0.6 ± 0.1 mU/ml in the mock-infected cultures (*P* = 0.039) (Fig. 2B). We did not find a significant difference in MPO in airway surface media from RSV-infected cultures following neutrophil migration for 4 h compared to the mock-infected cultures.

### 2.2 RSV infection results in increased neutrophil adherence to AECs

As we previously found that neutrophil adherence was associated with epithelial damage to RSV-infected ciliated cultures,^24^ here we also counted the number of neutrophils that migrate across and adhere to AECs using fluorescence microscopy. We detected no significant difference in the concentration of key neutrophil chemoattractant IL-8 at 24 h after RSV infection compared to the mock-infected control (Fig. 2C). However, the numbers of viable neutrophils adherent to AEC cultures infected with RSV for 24 h (791.1 ± 106.8 cells/cm^2^) or 72 h (711.1 ± 74.3 cells/cm^2^) were significantly (*P* = 0.014) greater than the respective mock-infected AEC cultures (449.8 ± 81.82 cells/cm^2^) or (486.6 ± 61.5 cells/cm^2^), respectively (Fig. 2D and E). Overall, transepithelial migration did not affect neutrophil viability (Fig. 2E). The total counts of viable neutrophils per well, i.e. combined basolateral, apically and adherent neutrophils, is shown (Fig. 2F).

At 72 h post infection, the concentration of neutrophil chemoattractant IL-8 and interferon gamma-induced protein 10 (IP-10) were significantly (*P* < 0.05) increased in RSV infected AECs compared to the mock-infected control cells (Fig. 2C). However, we found significantly (*P* = 0.006) fewer neutrophils remained adherent to mock-infected AECs exposed to RSV infected AEC supernatant (462.7 ± 43.3 cells/cm^2^), in comparison to the RSV-infected AECs (711.1 ± 74.3 cells/cm^2^) (Fig. 2D). This implies that neutrophil adherence is facilitated by RSV-infected epithelial cells, rather than soluble, secreted factors released by RSV-infected AECs into the supernatant. This was supported by our finding that RSV infection did not lead to an increase in the number of apical neutrophils (those that migrate and detach from the epithelial cells), with an average (mean ± SEM) of 22,876 ± 12,713 neutrophils/well, compared to the mock-infected AEC cultures (19,184 ± 10,806) at 24 h post infection (Fig. 2G). Significantly (*P* = 0.007) more apical neutrophils were recovered from mock-infected AECs exposed to RSV-infected AEC supernatant (RSV Sup), with an average 70,016 ± 21,115 neutrophils/well compared to the mock with 19,184 ± 10,806 and 22,876 ± 12,713 in comparison to RSV (*P* = 0.006) (Fig. 2G). These data were similar at 72 h postinfection (*P* = 0.980) for the number of neutrophils recovered from the RSV-infected AEC cultures (40,415 ± 15,143 neutrophils/well) compared to the mock (59,900 ± 18,885) (Fig. 2G).

### 2.3 Neutrophils upregulate expression of key surface markers following migration across AECs

So far, this study has shown that neutrophils are capable of trans-epithelial migration across airway epithelial cells and either “remain” on the *basolateral* side, become *adherent* to AECs, or migrate and dissociate into the *apical* space (see Fig. 1). To determine whether the ability of a neutrophil to migrate and adhere to epithelial cells is associated with the expression of specific cellular markers associated with neutrophil activation and migration (i.e. CD11b, CD64, CD62L, NE, MPO), we analyzed neutrophils recovered from basolateral, adherent, and apical compartments using flow cytometry. We chose the 72 h timepoint following AEC infection, and 1 h timepoint after neutrophil migration for these experiments as these were the conditions that resulted in the greatest number of adherent neutrophils (Fig. 2D) to allow effective comparisons.

**Fig. 3.**
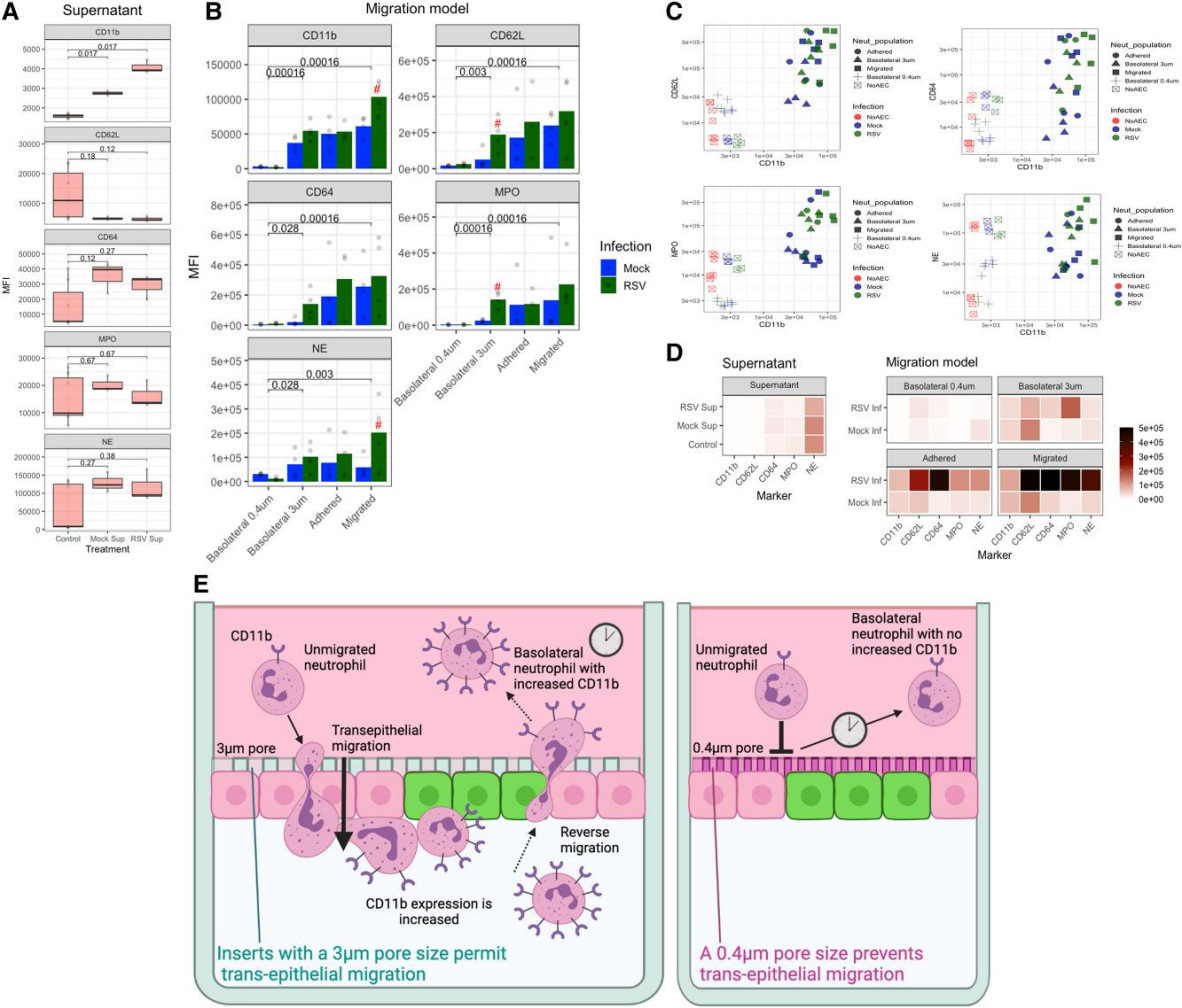
Neutrophils undergoing trans-epithelial migration alter their expression of surface markers depending on their location. (A) Mean fluorescence intensity (MFI) of cell surface expressed CD11b, CD64, CD62L, NE, and MPO on neutrophils incubated for 1 h with media alone (control) or media containing supernatant recovered from RSV- or mock-infected AECs. No airway epithelial cells were present in this model. Only a significant increase in CD11b was detected in this model between neutrophils exposed to RSV compared to mock-infected supernatant. (B) MFI of the same neutrophil markers as (A), but these were measured on neutrophils recovered after 1 h exposure to our airway epithelial migration model. Data show the MFI of markers on neutrophils recovered from the basolateral, adherent, and apical (migrated) compartments within the assay migration across mock- or RSV-infected AEC cultures. AECs were grown on membrane inserts with a 3 μm or 0.4 μm pore size; the latter prevents cellular migration (see Supplementary Fig. 1). *P* values show significant difference between 0.4 um model for RSV infection condition. Red # represents a significant (*P* < 0.05) difference between mock and RSV conditions for that neutrophil population using Wilcox-signed rank test, n = 4. (C) Correlation of CD11b expression with other markers measured showing clustering of migrated neutrophils in top right section of graph. (D) Heatmap summary of MFI of neutrophils exposed to supernatant alone or the migration model. A linear mixed-effects model was fitted to compare interactions between infection and location groups accounting for intra-donor variability. Individual comparisons performed with 2-way ANOVA with pairing and Tukey's post hoc test. * = in comparison to respective basolateral group; # = in comparison to respective adherent group. (E) Graphical illustration of possible interpretation of findings of CD11b expression. Left panel shows primary airway epithelial cells cultured at ALI on membrane inserts with 3 μm pore size that permits neutrophils to migrate through. (1) Unmigrated neutrophils expressing baseline levels of CD11b; (2) neutrophils migrate across infected AECs, and some remain adherent to the infected AECs; (3) neutrophils are shown to increase expression of CD11b and other activation-associated markers; (4) some “activated” neutrophils undergo reverse migration as (5) neutrophils with the increased expression of CD11b are detected on the basolateral side of the insert. Right panel shows primary airway epithelial cells cultured at ALI on membrane inserts with 0.4 μm pore size that prevents neutrophil contact with AECs and migration. Here, after 1 h incubation with RSV-infected AECs, neutrophils on the basolateral side (1) and (2) were shown to express the same level of CD11b markers, indicating that neutrophil contact with AECs and starting to move through the AECs is a key process for increasing expression of these activation markers. Drawing created using BioRender.com.

First, as a control, we determined whether naïve neutrophils altered their expression of CD11b, CD64, CD62L, NE, or MPO following exposure to epithelial-derived factors contained in apical supernatants recovered from mock- or RSV-infected AECs, but in the absence of AECs (Fig. 3A). Here, we found a significantly greater expression of CD11b on neutrophils incubated with RSV infected AEC supernatant (4,078.3 ± 109.8) compared to neutrophils incubated with media alone (1,583.7 ± 34.3) (*P* = 0.017) or mock-infected AEC supernatant (2,751 ± 37.5) (*P* = 0.017) (Fig. 3A). However, we found no significant difference in CD62L, CD64, NE, or MPO expression on neutrophils incubated with supernatants from mock- and RSV-infected AECs (Fig. 3A).

Introducing neutrophils to our migration model led to a significant increase in expression of CD11b and CD64 (*P* = 0.0002, *P* = 0.005, respectively) in neutrophils recovered from basolateral, adherent, and apical compartments of RSV-infected AEC models compared to neutrophils exposed to media alone (control) (Fig. 3B) (comparison not directly shown on graph). Interestingly, we detected a significant (*P* = 0.008) 24- and 30-fold increase (*P* < 0.001) in CD11b expression on neutrophils recovered from the basolateral compartments of both mock (36,708 ± 3,563) and RSV-infected AECs (54,389 ± 3,863) compared to neutrophils exposed to media alone (control) (1,583.7 ± 34.3) (Fig. 3B).

To determine whether this was due to factors secreted into the basolateral compartment by the AECs, and therefore independent of migration, AECs were grown on membranes with a 0.4 μm pore size. This smaller pore size does not permit neutrophils to move across the membrane and have contact with AECs (Supplementary Fig. 1) but will allow for passive diffusion of secreted factors. Using this system, we found that the expression of CD11b, CD64, CD62L, and MPO on basolateral neutrophils exposed to AECs grown on inserts with a 0.4 μm pore size did not increase and, in fact, was no different from the values obtained in neutrophils exposed to media alone (control). Compared to neutrophils recovered from the basolateral side of the RSV-infected AEC cultures grown on 3 μm inserts, neutrophils incubated on AECs grown on inserts with 0.4 μm pores demonstrated CD11b, CD64, CD62L, NE, and MPO levels that were at least 30× lower (Fig. 3B). This reduction in neutrophil activation was significant (*P* < 0.05) across all groups tested (mock and RSV infected), suggesting that the expression of these markers increases on neutrophils in the basolateral and adhered neutrophils because of direct contact with the AECs rather than infection (Fig. 3C). Following trans-epithelial migration, we found that RSV infection was associated with a further 1.5-fold increase (*P* = 0.021) in CD11b expression on apical (104,145 ± 6,631) neutrophils compared to neutrophils recovered from the respective compartments of mock-infected cultures (61,466 ± 4,876) (see Fig. 3B and summarized in Fig. 3E). There was no significant difference in CD64, CD62L, NE, or MPO expression on neutrophils recovered from RSV compared to mock-infected AECs (Fig. 3B). The highest expression levels for CD11b were recorded on apical neutrophils recovered from RSV-infected AECs (104,145 ± 6,631), which was more than 2-fold higher (*P* < 0.05) than both basolateral neutrophils (54,389 ± 3,863) and adherent neutrophils (53,561 ± 3,932) in the same model and >10,000-fold higher than neutrophils in media alone (control) (1,583.7 ± 34.3) (Fig. 3B and summarized in Fig. 3E), suggesting that both RSV infection and migration of neutrophils was associated with this high expression of CD11b. A graphical illustration of this is shown in Fig. 3E.

### 2.4 Temporal analysis of neutrophil trans-epithelial migration reveals that migrated neutrophils move more slowly but farther during RSV infection

We used time-lapse fluorescence microscopy imaging and performed vector analysis (or XYZ coordinates) of fluorescently labeled neutrophils to determine differences in the speed, track duration, distance traveled, and directionality of neutrophils (n = 200 per condition) during trans-epithelial migration through mock- and RSV-infected AECs.

#### 2.4.1 Speed

**Fig. 4.**
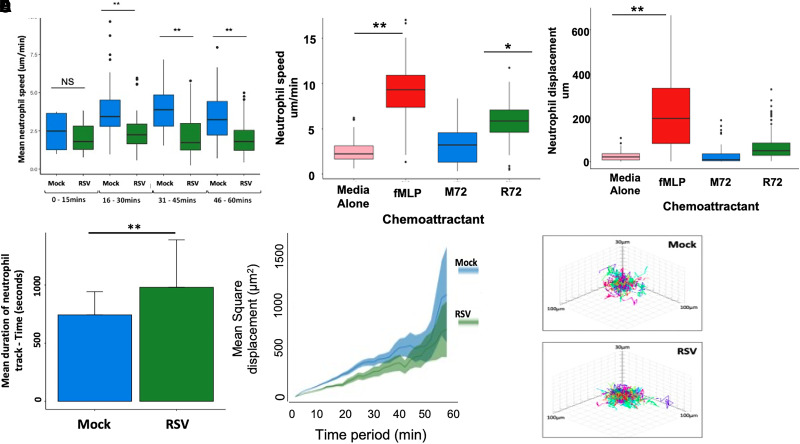
Neutrophil trafficking and directionality when exposed to supernatants from or RSV-infected AECs (green) or mock-infected AECs (blue) infected for 72 h. (A) Average neutrophil speed during trans-epithelial migration across mock- and RSV-infected AECs. This time series was split into 15-min segments. (B) 2D Chemotaxis in ibidi chemotaxis chambers showing the average speed neutrophils move in response to apical supernatants from AECs. (C) Neutrophil displacement—the gross distance traveled by individual neutrophils in response to apical supernatants from AECs. (D) Average track duration of 200 individual neutrophils during movement through mock- and RSV-infected AECs. Speed of tracks were calculated using motilityLab. (E) Mean squared displacement of neutrophils at each time point during movement across mock- and RSV-infected AECS. Statistical analysis between groups were performed using a Wilcoxon-Mann-Whitney *U* test; where significance was found, this is indicated on the chart. *** *P* < 0.001. F) Visualization of 50 tracks.

We found that, over the course of the first hour, neutrophils’ average (mean ± SEM) speed across RSV-infected AECs was significantly (*P* < 0.001) slower (2.28 μm/s ± 0.008) than the average speed of neutrophils moving through the mock infected AECs (4.18 μm/s ± 0.14) (Fig. 4A). The exception to this was the first 15 min of exposure, where we found no significant difference in speed of neutrophils migrating across RSV- and mock-infected AECs (Fig. 4A).

To determine whether this was associated with chemoattractants in the apical supernatant, we tracked the movement of naive neutrophils toward apical supernatants collected from RSV- or mock-infected AEC cultures using specialized 2D chemotaxis chambers (Fig. 4B). Here, we found that neutrophils exposed to supernatants collected from RSV-infected AEC cultures moved faster (0.205 μm/s ± 0.001) than neutrophils exposed to supernatant collected mock-treated AECs (0.110 μm/s ± 0.001) (*P* < 0.05) (Fig. 4B). At their fastest recorded speed (0.568 μm/s ± 0.049), neutrophils exposed to supernatants collected from RSV-infected AECs were 1.5 times faster (*P* = 0.018) than those migrating toward supernatant collected from mock-treated AECs (0.377 μm/s ± 0.014). There was no significant difference between the neutrophils exposed to media only.

#### 2.4.2 Duration

Measuring cumulative displacement (i.e. total distance traveled), we found that neutrophils exposed to supernatants collected from RSV-infected AECs moved significantly (*P* = 0.016) further (349 μm ± 49.9) than those exposed to media alone (182.7 μm ± 8.96) (Fig. 4C). We also measured the duration of neutrophil tracks during trans-epithelial migration (i.e. the length of time each neutrophil interacts with epithelium). Here we found that neutrophils interacted with RSV-infected AECs (368 μm ± 49.9) for longer than mock-infected AECS (Fig. 4D). We found no significant difference in linearity, or “track straightness,” between neutrophils observed migrating through RSV-infected AECs or the mock (Supplementary Fig. 2). However, neutrophils moving through mock-infected AECs showed significantly (*P* < 0.001) greater total displacement (137.19 μm ± 4.51) compared to those moving through RSV-infected AECs (107.05 μm ± 4.86) (Fig. 4E).

#### 2.4.3 Directionality

We performed some preliminary analysis of the Z-tracks of neutrophils (*n* = 20) migrating across RSV-infected AECs (3 representative tracks; Supplementary Fig. 3). This showed preliminary evidence that neutrophils may move bidirectionally across the AECs, migrating to the apical side of RSV-infected airway epithelium and returning to the basolateral compartment as soon as 15 min after migration. This supports the hypothesis that the increased CD11b expression in neutrophils in the basolateral compartment is due to a proportion of those neutrophils having undergone reverse migration.

### 2.5 Neutrophil clustering occurs 20 min after neutrophil recruitment

**Fig. 5.**
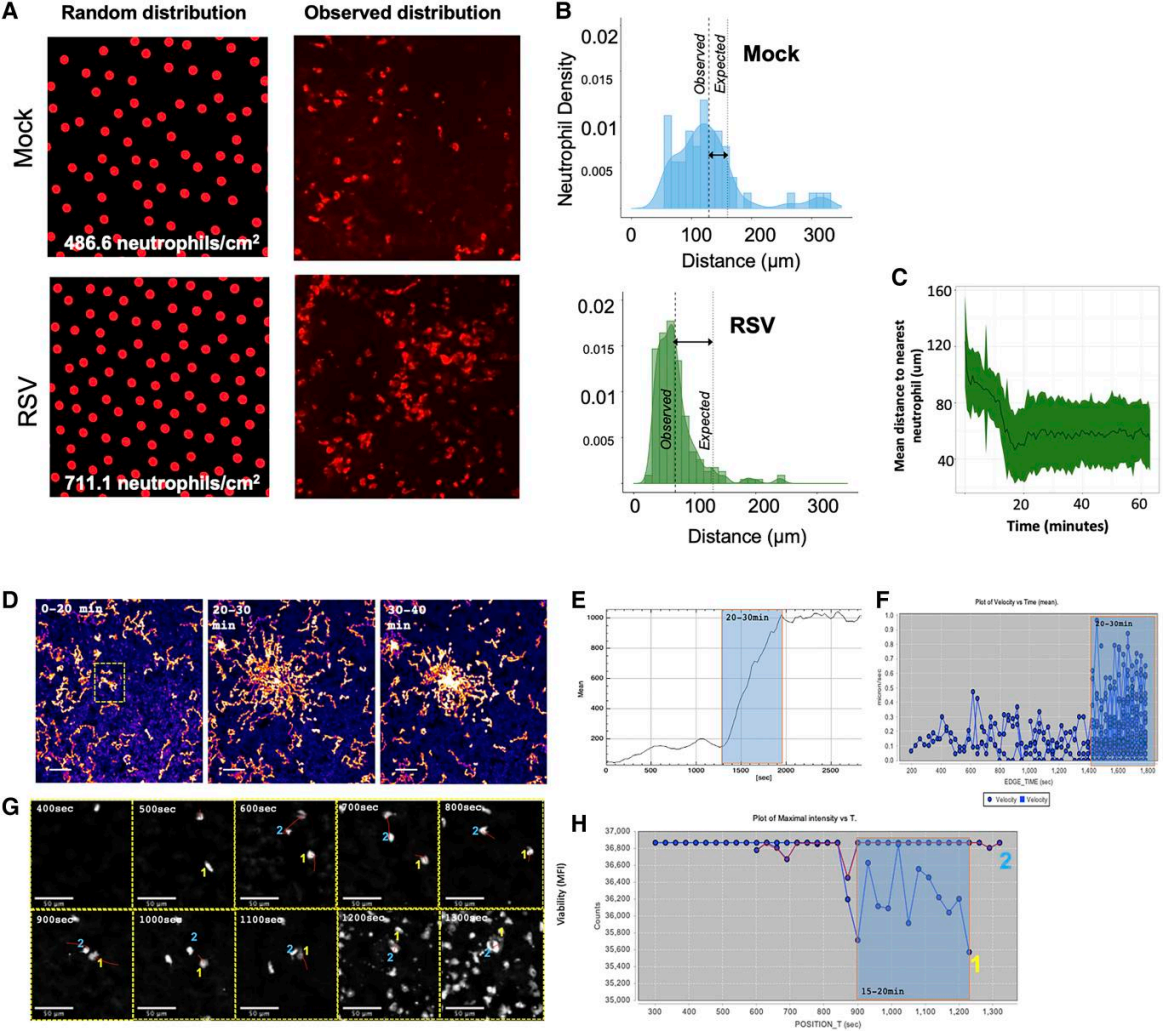
(A) Distribution analysis of neutrophils adherent to AECS infected with mock (left panel) and RSV (right panel) for 72 h following trans-epithelial migration. AECs were fixed after 1 h; neutrophil position was processed using ImageJ and coordinates analyzed using R version 4.0.3. (B) Histogram showing the frequency of neutrophils the distribution of distance to nearest neighboring neutrophil calculated from neutrophils adherent to the mock (blue) or RSV (green) infected AECs after 1 h. Thin dashed lines show median distance to nearest neighboring neutrophil. Thick dashed lines show median distance to nearest neighboring neutrophil assuming a uniform distribution of adherent neutrophil counts. Median distances were compared using a Wilcoxon-Mann-Whitney *U* test. (C) Average nearest neighbor distance of neutrophils over time during migration through RSV-infected AECs, calculated as above from fast time-lapse video microscopy (n = 200). Mean shown with dark green line. Range (min-max) indicated in color block. Track metrics calculated using motilitylab. Neutrophil swarming dynamics during RSV infection in a human model. (D) Time course of migration tracks on apical side of AEC following addition of neutrophils on basolateral side. Maximal intensity projection of the first 20-min (left panel) or 10-min (middle and right panels) time intervals. Scale bars, 100 μm. (E) Time course of neutrophil accumulation (by MFI) on the apical surface of RSV-infected AECs. (F) Time course of neutrophil accumulation by number of neutrophils on apical side per time interval is shown. (G) Images and MFI (H) of a single neutrophil (#1) becoming calcein red orange-negative at 15 min and its correlation to neutrophil-amplified chemotaxis (red tracks). Scale bars, 50 μm. Neutrophil positions were processed using ImageJ (imagej.nih.gov/ij/) and coordinates analyzed using R version 4.0.3.^28^

We previously observed that neutrophils adherent to RSV-infected epithelium formed large clusters.^24^ Here, we aimed to quantify this clustering and determine whether it was significantly enhanced during RSV infection. To do this, we first determined the expected nearest neighbor median distance, assuming an even distribution of the average number of adherent neutrophils. As more neutrophils are adherent to RSV-infected AECs compared to the mock-infected, we determined that the median distance of the *expected* nearest neighbor was shorter in RSV-infected AEC cultures (140 μm ± 24.3) in comparison to neutrophils adherent to the mock-infected epithelium (169.4 μm ± 57.8). The *observed* nearest neighbor median distance was also shorter in RSV-infected AEC cultures (61.32 μm ± 30.9) in comparison to neutrophils adherent to the mock-infected epithelium (119.4 μm ± 51.6) (Fig. 5B). Importantly, the observed distances between neutrophils adherent to RSV-infected AECs (61.32 μm ± 30.9) was significantly (*P* < 0.001) shorter than the expected distance (140 μm ± 24.3) (Fig. 5B). This indicates that the distribution of these adherent neutrophils on the epithelial surface is neither random nor uniform.

We then determined when, during trans-epithelial migration, neutrophils begin to cluster to RSV-infected AECs. This was defined as the earliest timepoint that the observed and expected nearest neighbor median distance became significantly different (Fig. 5C). We found that after 20 min, the observed median (± interquartile range [IQR]) difference between neutrophils and their nearest neighbors (102.9 μm ± 55.8) was significantly shorter (*P* < 0.001) than the distance expected by chance alone (140 μm ± 24.3) (Fig. 5C). This suggests that neutrophil clustering begins to occur around 10 min after the addition of neutrophils to the basolateral side of AECs in our model. Between 10 and 20 min, we detected a more marked rate of decline in nearest neighbor distance to 42.9 μm ± 55.8. After this time phase, and between 20 and 60 min, the mean distance to nearest neutrophil stays constant around 50 µm (Fig. 5C).

### 2.6 Location of neutrophil clustering is initiated by a dying neutrophil

To observe the initiation of neutrophil clustering to RSV-infected AECs, we used a higher-speed and resolution image capture system (see Supplementary Video 3). Here neutrophils, labeled with a fluorescent viability stain (calcein red-orange), were found to rapidly nucleate and form a cluster on the apical side of the AECs approximataely 20 min after the basolateral addition of neutrophils (Fig. 5D, E and F). This pattern of clustering bears resemblance to previous in vivo work, which described this observation as neutrophil swarming.^26^ Previous in vitro assays with bacteria have indicated that dying neutrophils precede the swarm-like formation of neutrophil clusters.^29^ To determine the probable source of the nucleation point in our model, we segmented a region of interest immediately adjacent to the cluster nucleus (shown in Fig. 5D) and tracked the neutrophils in the preceding 0 to 20 min. These data (Fig. 5G and H) show that the timing of the death (loss of viability stain) of a single neutrophil (labeled number 1 in Fig. 5G and H) correlated to the amplified recruitment of the neutrophil population at 20 min, which was directed toward the unviable neutrophil (see Supplementary Video 4). This indicates that neutrophil death may serve as a catalyst for swarming during RSV infection.

## 3 Discussion

This study has, for the first time, performed spatial, temporal, and functional analysis on migrating neutrophils in response to RSV infection of primary human airway epithelial cells. Our findings identify 3 putative phases of neutrophil migration:

1. Initial chemotaxis and adherence
2. Activation and reverse migration
3. Amplified chemotaxis and clustering

First, we found that RSV infection led to greater, but slower, net movement of neutrophils across RSV-infected AECs in comparison to mock-infected AECs. This contradicted with our findings that, in the absence of AECs, neutrophils moved faster toward RSV-infected supernatants (that contained elevated levels of the potent neutrophil chemoattractants CXCL8 (IL-8) and CXCL10 (IP-10) (Supplementary Fig. 2), compared to neutrophils exposed to mock-infected AEC supernatants. This suggests that an airway epithelial cell factor is responsible for slowing down the migration of neutrophils in the RSV-infected AEC model. This could be due to the interaction of epithelial ICAM-1 to neutrophil integrin LFA-1, as we have previously shown.^30^ Alternatively, cell syncytia formation, a known histopathological characteristic of RSV infection, could increase epithelial impedance by reducing the availability of accessible cell-cell junctions through which migration may occur.^30,31^ This may not only slow down the initial neutrophil response to RSV infection, but also prolong its duration and slow its resolution, contributing to a heightened period of inflammation.

Second, we found that trans-epithelial migration led to apical (but not basolateral) secretion of soluble neutrophil granular factors including MMP9, myeloperoxidase (MPO), and neutrophil elastase (NE) that correlated to epithelial damage. Migration also led to greater expression of neutrophil activation markers CD11b, CD64, CD62L, NE, and MPO, with the highest expression recorded on neutrophils recovered from the apical compartment of RSV-infected AECs. Interestingly, neutrophils recovered from basolateral compartments of the AEC model also increased expression of CD11b, CD64, CD62L, and MPO, but not NE, compared to the media and epithelial-derived supernatant controls. This is supported by clinical studies of children hospitalized with RSV bronchiolitis, which showed that neutrophils with upregulated CD11b were recoverable both from the airways by bronchiolar lavage and from peripheral circulation.^9^ Furthermore, neutrophils recovered from peripheral circulation of infants with RSV bronchiolitis have been shown to contain RSV mRNA.^11^ Viraemia due to RSV has not, to our knowledge, been observed clinically, which suggests that the virus causes symptoms because of its effects on the respiratory tract. This poses an important question: are activated neutrophils preferentially selected for migration, or does the process of trans-epithelial migration increase expression of neutrophils activation markers? To address this, we cultured our AECs on membrane inserts with a smaller pore size (0.4 µm), which prevented neutrophil migration. Here, following RSV infection, we did not find any difference in CD11b, CD64, CD62L, or MPO expression on basolateral neutrophils compared to neutrophils exposed to media alone. This suggests that neutrophils increase their expression of CD11b, CD64, CD62L, and MPO during or following migration, rather than an existing subpopulation of activated neutrophils being selected for migration. This was different for NE. Here we detected a decrease in NE expression when neutrophils were exposed to basolateral side of RSV-infected AECs, compared to the mock-infected. as a similar pattern in NE expression is seen when comparing neutrophils exposed to the no-AEC mock and RSV supernatants, suggesting that secreted factors may drive this change. Therefore, the presence of these “activated” neutrophils on the basolateral side of the AECs following migration may suggest that neutrophils, which are “activated” by the apical environment, reverse migrate to the basolateral side of AECs (see Graphical Abstract). To investigate this further, we analyzed the Z-tracks of neutrophils migrating across RSV-infected AECs (Supplementary Fig. 3). This showed that neutrophils can move bidirectionally across the AECs, quickly migrating to the apical side of RSV-infected airway epithelium and returning to the basolateral compartment as soon as 15 min after migration. This important finding may suggest that reverse migrating neutrophils have the ability to re-enter the peripheral circulation and contribute to vascular leakage or parenchymal necrosis, as seen in severe cases of RSV bronchiolitis.^32^

Next, we found that RSV infection led to greater numbers of neutrophils remaining adherent to RSV-infected AECs. This may be due to increased expression of host cell receptors including ICAM-1 (intercellular adhesion molecule 1), which we (and others) have previously shown to be upregulated in vitro airway models of RSV infection, that mediate neutrophil adherence to RSV-infected AECs.^24,30^ In support of this, we found that fewer neutrophils remained adherent to mock-infected AECs exposed to RSV-infected supernatant placed apically (in comparison to RSV-infected AECs). This suggests that properties of the epithelial cells, rather than the apical milieu of the RSV-infected epithelium per se, are responsible for neutrophil adherence. Interestingly, neutrophils were observed to adhere in clusters to the apical surface of RSV-infected AECs. Neutrophil clustering has been shown as a vital mechanism for host defense against pathogens.^22,24^ However, large clusters have been associated with inflammatory disease in both human and murine models.^33,34^ In models of RSV infection, increased neutrophil adherence and clustering have been associated with greater AEC damage.^23,24,35,36^

To examine the formation of these clusters in more detail, we used higher-resolution time-lapse microscopy to image the early movement of neutrophils moving across RSV-infected AECs. This showed that clusters can result from the coordinated convergence of neutrophils, which resembled reports of neutrophil swarming in response to infection and sterile injury in in vivo models.^24–26^ This pattern of neutrophil movement is thought to be initiated by the release of danger-associated molecular patterns (DAMPs) from neutrophils, which induces a transcriptional switch in their neighbors and coordinates a collective movement.^37^ LTB4 is a leukotriene DAMP, released by dying neutrophils, and it has been shown to mediate this directed leukocyte movement previously.^26,33,38,39^ We also showed that neutrophil death, or loss of cell viability, may serve as a catalyst for swarming during RSV infection. Here, we speculate that the formation of clusters of neutrophils, seen in our model, is not mediated by the RSV-infected AECs, but instead due to neutrophils themselves triggering and maintaining clustering behavior. The ability of neutrophils to affect others around them is an emerging area of research, and neutrophil quorum signaling has been shown to coordinate neutrophil collective responses to wound healing.^25,26,40^ Whether this response is a natural protective mechanism in countering an RSV infection or an aberrant response remains to be fully explained, but focal damage to AECs in vivo may allow increased neutrophil migration.

In summary, neutrophil activation has been shown as a key precursor to the development of severe respiratory symptoms in children with RSV bronchiolitis.^41^ Here we have shown that contact of neutrophils with AECs and/or trans-epithelial migration through RSV-infected AECs is essential for upregulation of neutrophil activation markers, including CD11b expression. We describe distinct, measurable patterns of neutrophil movement, including the formation of neutrophil clusters on RSV-infected AECs. We present evidence of the bidirectional movement of neutrophils across AECs during RSV infection and return of activated neutrophils to the basolateral side of infected AECs. This could explain how neutrophils with upregulated CD11b and the presence of viral products were recoverable in the blood neutrophils of babies with RSV bronchiolitis^9,11,25,37,40^ and could have important systemic implications for severe disease sequalae. This is a critical area for discovery, and the model that we have developed here could be used to unravel important disease mechanisms, including the key question of how RSV accesses extrapulmonary sites during infection. Future work should include the identification of new biomarkers of specific neutrophil subpopulations responsible for driving severe disease outcomes, screening anti-inflammatory drugs, and determining new mechanisms that may aid disease resolution.

## 4 Materials and methods

### 4.1 Participants

Peripheral blood and airway epithelial cells were obtained from healthy adult donors at UCL GOS Institute of Child Health. Written informed consent was obtained from all donors prior to their enrollment in the study. Study approval was obtained from the UCL Research Ethics Committee (4735/002). All methods were performed in accordance with the relevant guidelines and regulations.

### 4.2 Neutrophil isolation

Venous blood was collected in EDTA (Ethylene Diamine Tetra Acetic) tubes (Greiner). Neutrophils were then ultrapurified using an EasySep Direct Neutrophil isolation kit (Stem Cell Technologies) according to the manufacturer's instructions and subsequently stained with Cell Trace Calcein Red-Orange cell stain (ThermoFisher) and processed as described previously.^27^

### 4.3 Transepithelial migration model

This study modified the transepithelial migration model described by Herbert et al. (2020) to use undifferentiated human AECs, grown at an air–liquid interface for 7 d as opposed to 28-day ciliated cultures. This ensures a flatter, more uniform culture, which is possible to image using time-lapse confocal microscopy.^24^ AECs cells were cultured on porous PET inserts (Greiner), with a pore size of 3 μm to allow migration or 0.4 μm to prohibit it. AEC cultures were infected with RSV 24 or 72 h prior to the addition of neutrophils. Mock-infected AECs with RSV-infected AEC supernatant were used as a control (RSV Sup), as were mock-infected AECs with n-formylmethionine-leucyl-phenylalanine (fMLP) 100 nM placed apically as a positive control for neutrophil chemotaxis. 400 μl of supernatant was added underneath the membrane insert for each experimental group, mock, RSV, RSV supernatants only, and fMLP control. Excess supernatant prior to neutrophil migration was stored at −20 °C for use in chemotaxis and activation experiments. Neutrophils were then added to the basolateral side of all membrane inserts, and then left to incubate for 1 or 4 h. After migration, neutrophils were collected from the apical side of the epithelial cells for quantification. Supernatants were collected, and membrane inserts were fixed and stained. Mock infection was performed by inoculating with sterile media in the place of viral inoculum.

### 4.4 Virus purification and quantification

The recombinant GFP tagged RSV A2 strain was kindly provided by Jean-Francois Eleouet and described in Fix et al.^42^ Viral stock preparation and quantification of viral titer were performed using HEp-2 cells (ATCC CCL-23) as described previously.^24^

### 4.5 Microscopy

AEC cultures were fixed for microscopy using 4% paraformaldehyde (v/v) and mounted using 0.1 M n-propyll gallate in glycerol:PBS (9:1). Images of fixed cultures were acquired using an inverted Zeiss LSM 710 confocal microscope using a 20× Plan Achromat LWD objective. Live cell imaging was performed using a Perkin Elmer UltraView spinning disk (CSU22) confocal microscope. All microscopes were located in the UCL GOS Institute of Child Health Imaging Facility (London, UK).

### 4.6 Quantification of migrated and adherent neutrophils

The number of migrated neutrophils was quantified as described previously.^24^ Flow cytometric analysis of CD11b, NE, MPO, CD64, and CD62L expression was performed as described previously.^23^ Antibodies used are provided in the Supplementary Materials. Image acquisition was the same as above. Neutrophils were counted using an ImageJ counting tool.

### 4.7 Quantifying neutrophil distribution

To investigate whether neutrophil adherence to AECs was uniform (null hypothesis = 0) or whether they clustered, a method of measuring neutrophil distribution was required. Images of AECs after 1 h of migration were taken as described previously, and then analyzed using ImageJ to identify neutrophils within the image and determine their 3D coordinates. These coordinates were then exported into R and analyzed using the R package spatstat to then calculate the distance between the center-point of each neutrophil and the center-point of its 5 nearest neighbours.^43^ However, it is expected that if more neutrophils are adherent to RSV-infected AECs in comparison to mock, then they would be closer together by crowding alone. To account for this, the expected distance between each neutrophil, if neutrophils were evenly spread over the AEC area, was calculated from the numbers of neutrophils counted as adherent to the same AECs. The ImageJ macro code for determining 3D coordinates and subsequent R code for distance analysis is available upon request.

### 4.8 Analysis of neutrophil chemotaxis

Time-lapse videos were analyzed using Icy (https://icy.bioimageanalysis.org/) and programmed to run a spot detection protocol for each frame of the stack, detecting light objects close to 10 μm diameter on a dark background to identify neutrophils within the image. Then, a spot tracking plugin was used to map the displacement of each neutrophil through each time point image.^44^ The tracks produced were then visually checked for tracking accuracy and, where erroneous tracks were found, were corrected manually or removed from analysis. Raw spot files and track files were exported for further analysis using R. Data sets detailing total displacement, net displacement, average, maximum and minimum speed, and linearity were exported combined with replicate data sets for analysis using R and GraphPad Prism v.8.0.

### 4.9 Time-lapse imaging of neutrophil trans-epithelial migration in 4D (XYZT)

A black plastic 24-well plate with a glass coverslip bottom (Greiner) was used in place of a low binding 24-well plate (Greiner) to allow for live imaging of AEC cultures during trans-epithelial migration. Neutrophils were identified using Icy and a spot detection protocol initiated for each frame of the stack, detecting light objects close to 10 μm diameter on a dark background. Then each spot was tracked with the spot tracking plugin using a 4D tracking algorithm.^44^ Tracks were then visually checked for tracking accuracy, and where erroneous tracks were found, were either corrected manually or removed from analysis. Raw track coordinates were exported for further analysis using the R package motilitylab.^45^ Data detailing displacement, speed, and linearity were exported. Analysis of neutrophil movement and distribution was performed using the R packages motilitylab, spatstat, and dpylr.^46–48^

## Supplementary Material

qiad011_Supplementary_Data
